# Profiling Proteins and Phosphorylation Sites During T Cell Activation Using an Integrated Thermal Shift Assay

**DOI:** 10.1016/j.mcpro.2024.100801

**Published:** 2024-06-15

**Authors:** Brandon M. Gassaway, Edward L. Huttlin, Emily M. Huntsman, Tomer M. Yaron-Barir, Jared L. Johnson, Kiran Kurmi, Lewis C. Cantley, Joao A. Paulo, Alison E. Ringel, Steven P. Gygi, Marcia C. Haigis

**Affiliations:** 1 Department of Cell Biology, Harvard Medical School, Boston, Massachusetts, USA; 2 Department of Chemistry and Biochemistry, Brigham Young University, Provo, Utah, USA; 3 Meyer Cancer Center and Department of Medicine, Weill Cornell Medicine, New York, New York, USA; 4 Columbia University Vagelos College of Physicians and Surgeons, New York, New York, USA; 5 Dana Farber Cancer Institute, Boston, Massachusetts, USA; 6 Ragon Institute of Mass General, MIT, and Harvard, Cambridge, Massachusetts, USA; 7 Department of Biology, Massachusetts Institute of Technology, Cambridge, Massachusetts, USA; 8 Koch Institute for Integrative Cancer Research, Cambridge, Massachusetts, USA

**Keywords:** CD8^^+^^ T cell activation, proteomics, phosphoproteomics, proteome thermal stability, DNA repair, cyclin-dependent kinase signaling

## Abstract

T cell activation is a complex biological process of naive cells maturing into effector cells. Proteomic and phospho-proteomic approaches have provided critical insights into this process, yet it is not always clear how changes in individual proteins or phosphorylation sites have functional significance. Here, we developed the Phosphorylation Integrated Thermal Shift Assay (PITSA) that combines the measurement of protein or phosphorylation site abundance and thermal stability into a single tandem mass tags experiment and apply this method to study T cell activation. We quantified the abundance and thermal stability of over 7500 proteins and 5000 phosphorylation sites and identified significant differences in chromatin-related, TCR signaling, DNA repair, and proliferative phosphoproteins. PITSA may be applied to a wide range of biological contexts to generate hypotheses as to which proteins or phosphorylation sites are functionally regulated in a given system as well as the mechanisms by which this regulation may occur.

## Highlights

• T cells undergo significant proteome/phosphoproteome remodeling during activation.
• PITSA simultaneously measures abundance and thermal stability in a single TMT-plex.
• PITSA highlights abundance, signaling, and thermal stability changes in DNA repair.
• Increased CDK activity correlates with decreased CDK substrate thermal stability.

## Graphical Abstract

**Figure:**
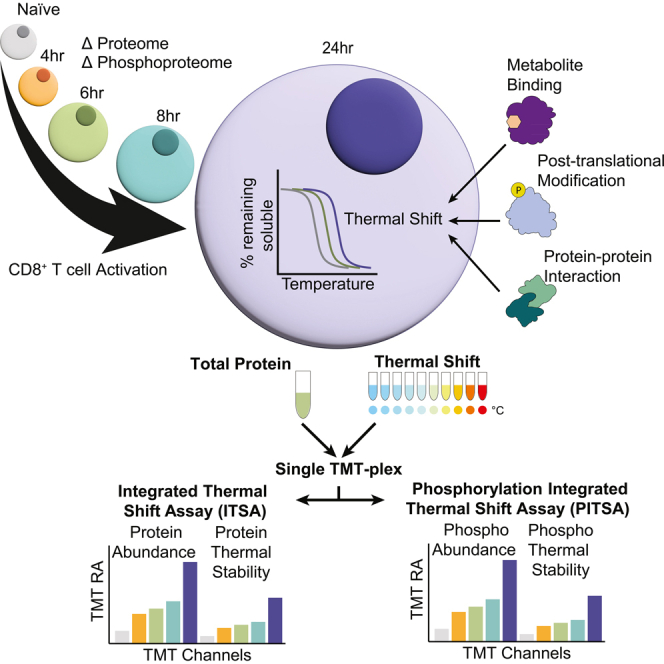

## In Brief

T cells undergo a significant rearrangement of their proteome and phosphoproteome during activation while accumulating necessary cellular components for rapid cell division. Here, we describe the phosphorylation integrated thermal shift assay, which combines the measurement of protein and phosphorylation site abundance with the measurement of protein and phosphorylation site thermal stability. This assay may help determine which proteome and phosphoproteome changes are functionally relevant in T cell activation by identifying proteins/phosphorylation sites with altered biophysical states, and presumably altered function.

Cytotoxic CD8^+^ T cells play a key role in the immune response against viral infections, autoimmunity, and cancer. Consequently, they are critical targets for therapy (*e.g.*, α-PD1) (1) and are therapeutic agents themselves (*e.g.*, CAR T cells) (2). Gaining insights into the mechanisms underlying the transition of these T cells from a naive, quiescent state to a state of growth and proliferation may enhance the precision and effectiveness of therapies and/or improve their effectiveness as therapies.

Within the initial 24 h of T cell receptor (TCR) engagement, T cells undergo substantial rearrangement of their proteome, phosphoproteome, and metabolism, priming them for clonal expansion (3, 4). These alterations are essential to generate material for rapid division and initiate the transition from a naïve T cell to an effector T cell. Numerous studies have examined these processes, including the delineation of early signaling events, and the identification of key regulatory nodes like mitochondrial biogenesis and one-carbon metabolism (5, 6). However, as T cell activation advances and the proteome changes, it is difficult to interpret how protein function is being regulated, as the majority of the proteome increases in abundance. Thus, changes in phosphorylation may be due to kinase activity or protein expression. Thus, novel techniques are necessary to deconvolute the relationship between protein phosphorylation and abundance.

**Fig. 1:**
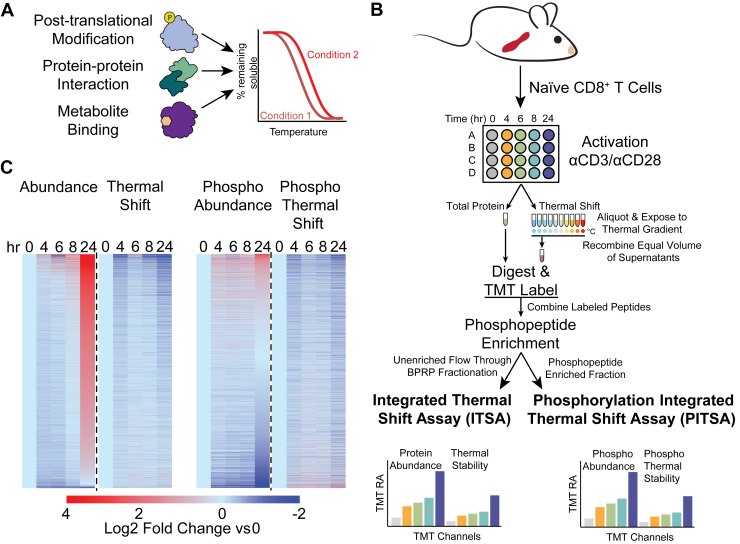
**The Phosphorylation Integrated Thermal Shift Assay.***A*, thermal stability as an intrinsic protein property can integrate many other inputs to protein regulation, including post-translational modification, protein-protein interactions, and protein-metabolite binding, which are measurable by a shift in the protein’s thermal melting curve. *B*, the PITSA workflow for investigating T cell activation. This method enables the simultaneous collection of four datasets (protein abundance, protein thermal shift, phosphorylation site abundance, and phosphorylation site thermal shift) in two sets of mass spectrometry experiments (protein abundance and thermal stability in one, phosphorylation site abundance and thermal stability in the other). *C*, heatmaps representing the four datasets (protein abundance, protein thermal shift, protein-normalized phosphorylation site abundance, and phosphorylation site thermal shift) collected for 5 time points across the first 24 h of T cell activation. Values are represented as the mean Log_2_ fold change *versus* 0 h (naïve) of n = 4 replicates per time point.

One recently developed technique for determining changes in protein function is by measuring protein thermal stability using mass-spectrometry-based proteomics (7, 8). To measure proteome thermal stability, these methods apply a temperature gradient to intact cells or cell lysates. When a protein reaches its melting temperature, it is thermally denatured, exposing its hydrophobic core which forms aggregates with other denatured proteins that are subsequently removed by centrifugation. The protein remaining in the solution is then digested and quantified by mass spectrometry. When the system is perturbed, a measurable shift in a protein’s thermal stability indicates a change in biophysical state and may indicate a change in regulation or function. This change in the biophysical state integrates changes in many other protein properties, such as post-translational modifications, protein-protein interactions, or protein-metabolite/small molecule interactions (Fig. 1*A*) (9, 10, 11, 12).

In this study, we sought to identify how protein thermal stability changes during T cell activation. We developed a pipeline to integrate adapted previous thermal shift assays by combining the protein abundance and thermal shift measurements into the same tandem mass tags (TMT)-plex in the Integrated Thermal Shift Assay (ITSA). Additionally, to elucidate how protein signaling corresponds to changes in thermal stability we also examined protein phosphorylation in the Phosphorylation Integrated Thermal Shift Assay (PITSA) (Fig. 1*B*). These innovations build upon previous thermal stability methods (7, 8, 12, 13, 14, 15, 16, 17, 18) and enable the simultaneous quantification of four biochemical properties at proteome scale: protein abundance, protein thermal stability, phosphorylation site abundance, and phosphorylation site thermal stability (Fig. 1, *B* and *C*). By measuring these four properties simultaneously using PITSA, we identified functionally meaningful changes in individual proteins and protein complexes, predicted functional phosphorylation sites by their altered thermal stability (and presumably function) compared to the bulk protein, and described the functional consequences of kinase activity. These results afford valuable insights into the molecular basis for T cell activation and demonstrate that PITSA is a useful strategy for probing proteome-level remodeling of complex biological systems.

## Experimental Procedures

### Experimental Design and Statistical Rationale

For the time course of T cell activation, an n of 4 was chosen as this number of replicates gives 70% power to detect at least a 2-fold change with *p* < 0.05 in a two-sided *t* test, and was experimentally tractable (pooling primary T cells from 5-6 mice gave sufficient starting material for both total protein and thermal stability measurements for 6 time points).

For the experiments investigating cyclin-dependent kinase (CDK) substrate thermal stability, n = 3 for each condition was chosen to fit into a TMT 18-plex experimental design (n = 3 each for DMSO, Nocodazole, and Nocodazole+CDKi; 9 samples for total protein and 9 for thermal stability).

### Mice

Wild-type C57BL/6J mice were purchased from the Jackson Laboratory (cat. #000664). Seven- to 11-week-old mice were used for all experiments. All experimental mice were housed in pathogen-free conditions at Harvard Medical School and were handled in accordance with approvals from the Harvard Medical School Institutional Animal Care and Use Committee, protocol # IS00000668-6.

### Primary Cell Culture

Naive CD8^+^ T lymphocytes were isolated from mouse spleens by negative selection using the naive CD8+ T cell Isolation Kit from Miltenyi Biotec (130-96-495). Following isolation, naive CD8^+^ T lymphocytes were activated with plate-bound anti-CD3 (4 μg/ml) (BioXCell, BE0001-1) and anti-CD28 (4 μg/ml) (BioXCell, BE0015-1) monoclonal antibodies. CD8^+^ T cells were cultured in RPMI-1640 supplemented with 10% heat-inactivated FBS, 10 mM HEPES (Gibco, 15630080), 0.05 mM 2-mercaptoethanol (Sigma, M3148-100 Ml), 1 mM Sodium pyruvate (Gibco, 11,360–070) and 1% penicillin-streptomycin.

### Jurkat Cell Culture

Jurkat T cells were cultured in RPMI-1640 supplemented with 10% heat-inactivated FBS and 1% penicillin-streptomycin. Cells were treated with either DMSO or nocodazole 1 μM overnight and then treated with CDK inhibitors Dinaciclib and Palbociclib at 1 μM for an additional 1 h. Cells were washed with 1xPBS, trypsinized, and counted. An equal number of cells were aliquoted for the PISA experiment.

### Flow Cytometry

Primary mouse CD8^+^ T cells were stained with fluorescent antibodies and analyzed by flow cytometry. Cells were first stained with LIVE/DEAD Fixable Near-IR stain (ThermoFisher Scientific, L4975) in 1× DPBS according to the manufacturer’s instructions. Surface marker staining was performed in MACS buffer containing 1× DPBS supplemented with 1% FBS and 2 mM EDTA. Samples were incubated for 30 min at 4 °C during surface staining using a cocktail of the following antibodies and dilutions: FcX Block (1:50, Biolegend, 101320), CD3 (1:100, BD Biosciences, 563565), CD8b (1:100, Biolegend, 126629), CD62L (1:100, Biolegend, 104408), CD44 (1:100, Biolegend, 103012), and CD69 (1:100, Biolegend, 104522). Data collection was performed on an LSR II flow cytometer and analyzed using FlowJo v10.4.1.

### Proteomics and Phosphoproteomic Sample Preparation of Primary and Jurkat T Cells

After washing cells 1× with PBS, a proteome integral solubility-like assay was performed as previously described (13). Briefly, cells were counted and diluted to 2.5 × 10^6^ cells/ml in PBS. 200 μl of cells were pelleted, supernatant removed and frozen in liquid nitrogen for the total protein/phosphorylation measurements, while 30 μl were aliquoted into each of 10 PCR tubes (total 300 μl) for PISA and exposed to a thermal gradient of 48 to 58 °C, after which 1× volume of lysis buffer (1% NP-40 in PBS with protease and phosphatase inhibitors (Pierce A32959)) was added, and incubated for 15 min while shaking at 4 °C. Insoluble aggregates and cell debris were pelleted by centrifugation at 21,000*g* for 90 min, and equal volumes of supernatant from each of the 10 PCR tubes were recombined into a single tube and frozen in liquid nitrogen. After thawing on ice, the 200 μl cell pellets were lysed with 100 μl of lysis buffer (0.5% NP-40 in PBS) and clarified by centrifugation, while the PISA samples were thawed on ice. 1× volume of PISA preparation buffer (400 mM EPPS, 1% SDS, 10 mM TCEP) was added and incubated for 10 min, followed by alkylation of cysteines by the addition of 10 mM IAA for 30 min in the dark, and quenching with 10 mM DTT for 10 min. Samples were desalted using the SP3 method (19), using magnetic beads (Cytiva 45152105050250 and 65152105050250), by incubating beads, sample, and 2× volume 100% ethanol for 15 min, followed by three washes with 80% ethanol.

Proteins were digested overnight at 37 °C while shaking in 200 mM EPPS pH 8.5 with 1 μg each of LysC (Wako 129-02541) and trypsin (Pierce 90305). Peptides were labeled by adding anhydrous acetonitrile (Honeywell AS017-0100) to ∼30%, followed by TMTpro (Thermo A44520) reagent. 1% of each labeled sample was combined and analyzed unfractionated to ensure labeling efficiency was >97%, and all channels were combined. After mixing, labeled peptide samples were de-salted using a 200 mg Sep-Pak cartridge (Waters WAT054925), followed by drying in a rotary evaporator. Phosphopeptides were enriched using a Fe-NTA spin column (Thermo A32992) and desalted *via* StageTip for LC-MS/MS analysis, while the flow-through was dried, reconstituted in 5% ACN 10 mM ammonium bicarbonate, and fractionated using basic pH reversed-phase chromatography on an Agilent 300extend-C18 column (3.5 μm, 4.6 × 250 mm) using an Agilent Infinity 1260 HPLC. Peptides were subjected to a 75 min linear gradient from 13% to 42% of Buffer B (10 mM ammonium bicarbonate, 90% ACN, pH 8) at a flow rate of 0.6 ml/min, resulting in a total of 96 fractions which were consolidated into 24 by combining (in a chessboard pattern) four alternating wells down columns of the 96-well plate as previously described (20). Each combined fraction was desalted *via* StageTip for LC-MS/MS analysis.

### Proteomic Data Collection

Mass spectra were collected on an Orbitrap Eclipse or Fusion Lumos mass spectrometer (ThermoFisher Scientific) coupled to a nanoEASY-nLC 1200 LC pump (ThermoFisher Scientific). Peptides were separated on a 35 cm column (i.d. 100 μm, Accucore, 2.6 μm, 150 Å) packed in-house using a 90 min gradient (from 5% −30% acetonitrile with 0.1% formic acid) at 500 nl/min. Peptide/protein data were collected using a Real-time search (RTS) (21, 22) SPS-MS3 (23, 24) method, while phosphopeptide data was collected using a high-resolution MS2 method. Using the SPS-MS3 method takes advantage of the increased quantitative accuracy, while RTS helps maintain proteome depth by minimizing the number of time-expensive MS3 scans. However, because this capability does not yet work for PTM searches we elected to use an MS2 method for phosphopeptide data collection (see below). For peptides/proteins, MS1 data were collected using the Orbitrap (120,000 resolution; maximum injection time 50 ms; AGC 4e5, 400–1400 m/z). Determined charge states between 2 and 5 were required for ms/ms and a 120s dynamic exclusion window was used. MS2 scans consisted of collision-induced dissociation (CID), quadrupole ion trap analysis, AGC 2E4, NCE 35, q-value 0.25, maximum injection time 35 ms, and isolation window of 0.7 Da using a Top10 method. Through Thermo Fisher Scientific’s instrument application-programming interface (iAPI) for Tribrid mass spectrometers, an online real-time search algorithm (using the UniProt Mouse database, 1/26/2022, 63,780 sequences including contaminants) was used to trigger MS3 scans for quantification (21, 22). MS3 scans were collected in the Orbitrap at a resolution of 50,000, NCE of 65%, maximum injection time of 100 ms, and AGC of 1.5e5. For phosphopeptides, MS1 data were collected using the Orbitrap (120,000 resolution; maximum injection time 50 ms; AGC 4e5, 400–1400 m/z). Determined charge states between 2 and 5 were required for sequencing and a 120 s dynamic exclusion window was used. MS2 scans consisted of higher collision energy dissociation (HCD) followed by Orbitrap analysis, with a resolution of 50,000, automatic gain control (AGC) 1.5E5, NCE (normalized collision energy) of 36, q-value 0.25, maximum injection time 250 ms, and isolation window of 0.5 Da using a Top10 method.

### Mass Spectrometry Data Processing

Mass spectra were processed using a COMET (25)-based (version 2020.01 rev. (4)) software pipeline. Data were searched against the UniProt (26) Mouse (1/26/2022, 63,780 sequences) or Human (11/24/2021, 101,173 sequences) database. For protein measurements, data were searched with a 20-ppm precursor ion tolerance and 1.0005 Da product ion tolerance with 0.4 Da fragment bin. TMTpro and carbamidomethylation of cysteine were set as static modifications, while methionine oxidation and asparagine/glutamine deamidation were set as variable modifications. A minimum peptide length of 7 and up to 2 miss cleavages were allowed. Peptide-spectrum matches (PSMs) were identified, quantified, and filtered to a 1% peptide false discovery rate (FDR) and then collapsed further to a final protein-level FDR of 1%. Proteins were quantified by summing reporter ion counts across all matching PSMs. Briefly, a 0.003 Da (3 mDa) window around the theoretical m/z of each reporter ion was scanned and the maximum intensity nearest the theoretical m/z was used. Reporter ion intensities were adjusted to correct for the isotopic impurities of the different TMT reagents according to manufacturer specifications and adjusted to normalize ratios across labeling channels. Lastly, for each protein, signal-to-noise (S:N) measurements of the peptides were summed and then normalized to 100. For phosphopeptide measurements, a 20-ppm precursor ion tolerance and 0.02 Da fragment ion tolerance were used, with TMTpro and carbamidomethylation of cysteine as static modifications, and methionine oxidation, asparagine/glutamine deamidation, and serine/threonine/tyrosine phosphorylation as variable modifications. Peptide-spectrum matches (PSMs) were identified, quantified, and filtered to a 1% peptide FDR. Phosphorylation sites were localized using AScorePro (27). Phosphorylation sites were quantified by summing reporter ion counts across all matching PSMs. Briefly, a 0.003 Da (3 mDa) window around the theoretical m/z of each reporter ion was scanned and the maximum intensity nearest the theoretical m/z was used. Reporter ion intensities were adjusted to correct for the isotopic impurities of the different TMT reagents according to manufacturer specifications and adjusted to normalize ratios across labeling channels. Lastly, for each phosphorylation site, signal-to-noise (S:N) measurements of all phosphopeptides with the same phosphorylation site were summed and then normalized to 100. Initially, a 2 h time point was included, however, we observed that many proteins in the thermal shift were below our limit of quantitation, and we removed this time point from future analyses (Supplemental Fig. S1, *G* and *H*). Phosphorylation site data (scaled sum S:N) was normalized to protein data (scaled sum S:N). The mass spectrometry proteomics data have been deposited to the ProteomeXchange Consortium *via* the PRIDE (28) partner repository with the dataset identifiers PXD050772 (T cell activation) and PXD050445 (Jurkat Cells). Data were analyzed using Perseus 2.0.6.0 (29), R (30) (including the Timecourse package (31)), and GraphPad Prism 9.5.0.

### BioPlex Network Analyses

Network analyses were performed by mapping protein subsets identified *via* ITSA and PITSA to the BioPlex 3.0 interaction network (Huttlin *et al*. 2021) after combining all interactions identified in both 293T and HCT116 cells. Mouse proteins were mapped to their human counterparts according to Entrez Gene ID according to homology lists maintained by Jackson Labs (reference website). All calculations were performed and figures were made using Mathematica 13.2 (Wolfram Research).

**Fig. 2:**
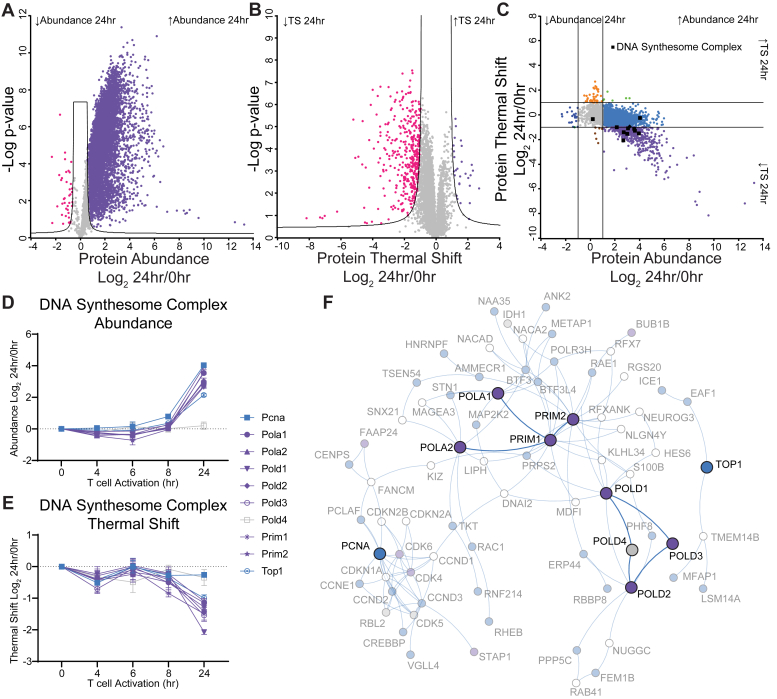
**ITSA Identifies Coordination in Thermal Stability Regulation Between Proteins and Protein Complexes.***A*, volcano plot comparing the Log_2_ fold change in protein abundance between 0 h and 24 h of activation. *B*, Volcano plot comparing the Log_2_ fold change in protein thermal shift (TS) between 0 h and 24 h. *C*, plotting the protein thermal shift *versus* the protein abundance separates proteins into categories of behavior. Lines represent 2-fold change. Members of the DNA synthesome complex are indicated by *black* squares and *black* diamonds, respectively. Changes in protein abundance (*D*) or thermal shift (*E*) for members of the DNA synthesome complex. Colors are based on category in (*C*). *F*, potein-protein interaction network of the DNA synthesome proteins and nearest neighbors from Bioplex 3.0, coloring as in (*C*). Values are the mean Log_2_ fold change of the indicated timepoint *versus* 0 h of n=2 to 4 replicates. Error bars (*D* and *E*) represent SEM.

**Fig. 3:**
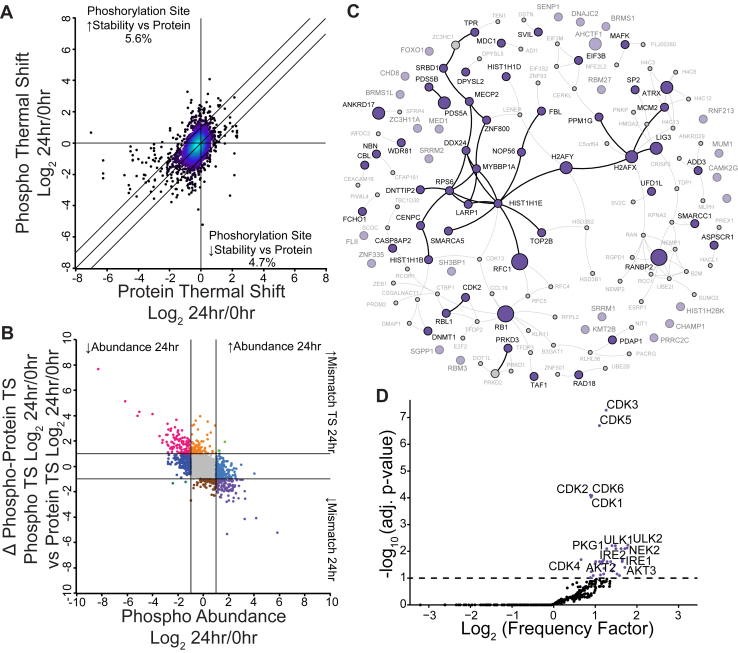
**PITSA Identifies Phosphorylation Sites that Alter Protein Thermal Stability.***A*, a comparison of the phosphorylation site thermal shift at 24 h *versus* the bulk protein thermal shift at 24 h. Lines indicate a 2-fold difference between protein and phosphorylation site thermal stability. *Darker colors* indicate reduced point density. Values are the mean Log_2_ fold change of 24 h *versus* 0 h of n = 4 replicates per time point. *B*, a comparison of the difference between phosphorylation site and protein thermal shift (Δ Phospho-Protein TS) *versus* phosphorylation site abundance separates phosphorylation sites into behavioral categories. Lines represent 2-fold change. *C*, the Bioplex network of co-regulated phosphorylation sites with increased abundance and decreased thermal stability. The size of the *circle* represents the number of phosphorylation sites from that protein observed in the category. *D*, a kinase motif analysis of phosphorylation sites changing in both abundance and thermal stability at some point in the time course.

After each protein subset was mapped to the BioPlex network, network propagation was used to identify other proteins that closely associated with the target protein class. Each protein in the target protein class was initially assigned a weight of 1.0. This weight was then propagated throughout the network *via* a random walk with a restart probability of 0.5 as described previously (32). Propagation was stopped after 40 steps and the final weights of each node in the network were recorded. Subsequently, this process was repeated for 100 randomized interaction networks whose structures were derived by scrambling the edges in the original BioPlex network while holding interaction counts constant for every individual node and maintaining the overall degree distribution. Network propagation scores from these randomized networks were used to derive averages and standard deviations and calculate Z-scores for each protein reflecting the extent to which the weights observed in the real network differ from those observed in the randomized networks. To generate Figures 2*F*, 3*C*, and Supplemental Fig. S3, nodes with Z-scores greater than 10 were selected for plotting, along with all proteins in the original target set. Figures were simplified by removing peripheral proteins that were not quantified *via* ITSA/PITSA and which connected to the rest of the subgraph by just a single interaction.

### The Kinase Library Enrichment Analysis

Kinase enrichment was performed based on the list of differentially phosphorylated sites described above. A full description of the substrate specificities atlas of the Ser/Thr kinome can be found in Johnson, *et al*. 2023) (33). The phosphorylation sites detected in this study were scored by all kinase position-specific scoring matrices (303 total). For each site, this score was compared to the respective phosphoproteome score distribution for each kinase to calculate the substrate’s percentile. This was subsequently used to rank the kinases. For every singly phosphorylated site, kinases that ranked within the top 15 (out of 303 S/T) were considered favorable for that phosphorylation site. To assess kinase motif enrichment between conditions, we compared the percentage of phosphorylation sites favored by each kinase within the upregulated sites (logFC ≥ 1) to the percentage of favored phosphorylation sites within the set of unregulated sites in this study (−1<logFC<1). The corresponding comparison was also made between downregulated (logFC<=-1) and unregulated ((-1<logFC<1) phosphorylation sites. Contingency tables were corrected using Haldane correction. Statistical significance was determined using a one-sided Fisher’s exact test, and the corresponding *p*-values were adjusted using the Benjamini-Hochberg procedure. Then, for every kinase, the most significant enrichment direction (upregulated or downregulated) was selected based on the tests’ resulting adjusted *p*-values and presented in the volcano plots and bubble maps. In the volcano plots, kinases that were significant (adjusted *p*-value ≤ 0.1) for both upregulated and downregulated analysis were plotted on both sides and marked in yellow. Bubble maps were generated with size and color strength representing the adjusted *p*-values and frequency factors respectively, only displaying significant kinases (adjusted *p*-value ≤ 0.1). Kinases that were significant (adjusted *p*-value ≤ 0.1) for both upregulated and downregulated analysis were plotted using the parameters of the more significant direction but were also outlined in yellow.

Key resources are described in Table 1.

**Table 1:** Key Resources Table

Reagent or Resource	Source	Identifier
Antibodies		
α-CD3	BioXCell	Cat#: BE0001-1
α-CD28	BioXCell	Cat#: BE0015-1
FeX Block	Biolegend	Cat#: 101320 RRID:AB_1574975
CD3	BD Biosciences	Cat#: 563565 RRID:AB_2738278
CD8b	Biolegend	Cat#: 126629 RRID:AB_2800620
CD62L	Biolegend	Cat#: 104408 RRID:AB_313095
CD44	Biolegend	Cat#: 103012 RRID:AB_312963
CD69	Biolegend	Cat#: 104552
Chemicals, Peptides, and Recombinant Proteins		
Protease and Phosphatase Mini Tablets	Thermo Scientific	Cat#: A32959
Bond-Breaker TCEP	Thermo Scientific	Cat#: 77720
Iodoacetamide	BeanTown Chemical	Cat#: 122600
Dithiolthreitol	Thermo Scientific	Cat#: R0862
LysC	Wako	Cat# 129-02541
Trypsin, MS Grade	Thermo Scientific	Cat#: 90305
Anhydrous Acetonitrile	Honeywell	Cat#: AS017-0100
TMTpro 16-plex reagent	Thermo Scientific	Cat#: A44520
TMTpro 18-plex reagent	Thermo Scientific	Cat#: A52046
Nocodazole	Sellekchem	Cat#: S2775
Dinaciclib	Sellekchem	Cat#: S2768
Palbociclib	Sellekchem	Cat#: S4482
Critical Commercial Assays		
LIVE/DEAD Fixable Near-IR Stain	Thermo Scientific	Cat#: L4975
Pierce BCA Assay	Thermo Scientific	Cat#: 23225
Deposited Data		
Identifying Functionally Relevant Proteins and Phosphorylation Sites During T Cell Activation Using an Integrated Thermal Shift Assay	PRIDE	PXD050772
Investigation of CDK substrate thermal stability in Jurkat T cells	PRIDE	PXD050445
Experimental Models: Cell Lines		
Jurkat	ATCC	Cat#: TIB-152
Experimental Models: Organisms/Strains		
C57BL/6J	Jackson Labs	Cat#: 000664 RRID:IMSR_JAX:000664
Software and Algorithms		
FlowJo v10.4.1	BD Biosciences	https://www.flowjo.com/ RRID:SCR_008520
Comet v 2020.01 rev. (4)	Eng, *et al.* 2013 (25)	https://uwpr.github.io/Comet/
Perseus v 2.0.6.0	Mann and Cox, 2016 (29)	http://www.coxdocs.org/doku.php?id=perseus:start RRID:SCR_015753
R	R Core Team (30)	https://www.r-project.org/ RRID:SCR_001905
Timecourse	Tai *et al.*, 2007 (31)	https://bioconductor.org/packages/release/bioc/html/timecourse.html RRID:SCR_000077
Mathematica 11	Wolfram	https://www.wolfram.com/mathematica/ RRID:SCR_014448
GraphPad Prism	Graphpad	https://www.graphpad.com/ RRID:SCR_002798
Other		
Naïve CD8^+^ T cell Isolation Kit	Miltenyi Biotec	Cat#: 130-96-495
High Select Fe-NTA Phosphopeptide Enrichment Kit	Thermo Scientific	Cat#: A32992
Sera-Mag SpeedBeads	Cytiva	Cat#: 45152105050250
Sera-Mag SpeedBeads	Cytiva	Cat#: 65152105050250
SepPAK C18 Cartridge	Waters	Cat#: WAT054925

## Results

### The PITSA

To identify functionally regulated proteins and phosphorylation sites during T cell activation, we isolated naïve CD8^+^ T cells from mouse spleen and activated them with anti-CD3/anti-CD28 monoclonal antibodies (mAbs) for 0, 4, 6, 8, and 24 h (Fig. 1, *B* and *C*). As T cells underwent activation, they exhibited increased cell size, sustained viability, and expressed activation markers as expected (Supplemental Fig. S1, *A*–*E*). At each time point, the cells were harvested and divided; part of the sample was preserved for abundance measurements while the remainder was subjected to a 48 to 58 °C temperature gradient (13). After thermal exposure, insoluble aggregates were removed by centrifugation, supernatants were recombined into a single sample which, along with the sample set aside for abundance measurements, was digested, labeled with TMT, and enriched for phosphorylated peptides. After enrichment, both the phosphorylation-enriched and non-phosphorylated flow-through fractions were analyzed using LC-MS. We refer to the approach for monitoring protein thermal stability as ITSA, while the integration of thermal stability with phosphorylation is referred to as PITSA.

Overall, we observed that nearly every protein measured shows a sharp increase in abundance at 24 h of activation, as reported previously (Fig. 1*C*, Supplemental Fig. S1*F*, and Supplemental Dataset S1) (5). However, even though shifts in thermal stability were widespread, they affected a smaller number of proteins overall than those with differential abundance and also tended towards thermal destabilization (Fig. 1*C* and Supplemental Fig. S2, *A* and *B*). These thermal shifts suggest additional layers of regulation beyond abundance, possibly from an alteration in the composition of proteoforms with distinct thermal stabilities within the population or from changes in protein-protein interaction networks (12).

The observation that thermal stability reports on additional layers of regulation remained true for the protein-normalized phosphorylation site data (Fig. 1*C* and Supplemental Fig. S2, *C* and *D*). Similar to the protein thermal shift, a smaller number of phosphorylation sites had altered thermal shifts compared to altered abundance, and they trended towards thermal destabilization (Supplemental Fig. S2, *C* and *D*). However, unlike the protein abundance, the phosphorylation site abundance trended toward decreasing, especially at the 24 h time point. Principal component analysis of the time points demonstrated clear clustering of replicates, and logical progression across the time course of T cell activation (Supplemental Fig. S1, *I*–*M*).

### ITSA Identifies Functionally Regulated Proteins and Complexes

To test if we could identify functionally regulated proteins using ITSA, we compared the fold-change in protein relative abundance for each time point *versus* naive T cells (Fig. 2*A* and Supplemental Fig. S2*A*), to the protein thermal shift for each time point *versus* naïve T cells (Fig. 2*B* and Supplemental Fig. S2*B*). In this context, changes in protein abundance reflect both protein synthesis and degradation; as we observed 6410 proteins significantly upregulated at 24 h *versus* 38 significantly downregulated when compared to the naïve state, it is clear that protein synthesis is the predominant paradigm (Fig. 2*A*). In contrast, thermal shifts represent alterations in the composition of protein, including (but not limited to) the ratio of bound to unbound protein, changes in protein localization, and/or the presence of post-translational modifications. At 24 h of activation, we observed only 28 proteins that were thermally stabilized *versus* 390 that were thermally destabilized. By comparing the change in abundance to the thermal shift (Fig. 2*C*), we separated protein behavior into functionally meaningful categories (34) using 2-fold change cutoffs in both dimensions. For example, contained in the upper central (orange) category are proteins whose abundance was not altered, but whose thermal shift was significantly increased; this category is enriched in chromatin-related proteins such as H2ax and hist1h1 (Supplemental Fig. S3 and Supplemental Table S1). Contrasting those proteins are the center-left and center-right (light and dark blue) categories where proteins were only altered in abundance and not thermal stability.

Next, we identified proteins that increased in abundance but decreased in thermal stability (lower right [purple] category). We observed that many members of the DNA synthesome complex were included in this category. Most members of the complex-except POLD4-are regulated similarly in abundance, while most-except for POLD4 and PCNA-are regulated similarly in thermal shift (Fig. 2, *D* and *E*). This observation correlates with the BioPlex protein-protein interaction network of this complex (35, 36, 37), in that there is some distance between the two core subnetworks (containing the PolA-Primase complex and the PolD complex) and PCNA, suggesting that even though PCNA is a member of the synthesome complex the bulk of it may associate elsewhere (Fig. 2*F*). Interestingly, recent studies have shown that the PolD subcomplex can exist in a tetrameric form, involved in progressive DNA synthesis, and a trimeric form without POLD4, which performs more of a proofreading and DNA editing function (38, 39); our abundance and thermal stability data indicate that the latter, trimeric complex is the predominant functional complex in this experimental regime, suggesting a DNA damage phenotype during this timeframe of T cell activation. Indeed, supporting this observation is the fact that DNA repair is among the GO terms enriched in the increased abundance/decreased thermal shift category (Supplemental Table S1).

### PITSA Identifies Functionally Regulated Phosphorylation Sites

Using PITSA, we also identified functionally relevant phosphorylation sites by comparing the phosphorylation site fold change to the bulk protein fold change (Fig. 3*A* and Supplemental Fig. S4*A*). Phosphorylation sites within the y = x ± 1 central stripe have less than a 2-fold change difference between the phosphorylation site and bulk protein thermal stability; however, we identified 572 phosphorylation sites that have at least a 2-fold difference in thermal shift compared to the bulk protein in at least one time point. When comparing the 24 h time point to naïve T cells, 5.6% and 4.7% of phosphorylation sites were more or less thermally stable than the bulk protein, respectively. These sites indicate a separate population of protein that has an altered biophysical state, with a presumed altered function. We next compared the difference between the phosphorylation site and protein thermal shifts (Δphospho-protein TS) to the difference in phosphorylation site abundance to categorize phosphorylation site behavior (Fig. 3*B*). Initially, we hypothesized that because 14-3-3 proteins are known to bind phosphorylated residues, that we would observe differences between phosphorylation site and protein thermal stability at 14-3-3 sites. Yet, the data did not support this idea, as we generally did not see phosphorylation site-protein thermal shifts for predicted 14-3-3 binding sites (Supplemental Fig. S4*B*). However, we did observe other phosphorylation sites with thermal shifts; amongst phosphorylation sites that have increased abundance and reduced thermal stability compared to the bulk protein, we observe a coregulated protein interaction network consisting of many nuclear, chromatin, and cell cycle-related proteins (Fig. 3*C*). Interestingly, these phosphorylation sites include those on H2AX and HIST1H1, which are correlated with the observation that these proteins had altered thermal stability without changes in abundance.

In addition to identifying functionally relevant phosphorylation sites, we tested whether these phosphorylation sites were changed due to altered protein abundance (similar ratio of phosphorylation site to protein), or whether the site had an altered stoichiometry (an altered ratio of phosphorylation site to protein) by comparing the ratio of Hotelling T (2) statistics for phosphorylation site abundance and protein abundance to the change in phosphorylation site abundance (Supplemental Fig. S5, *A* and *B*) (34). Hotelling T (2) statistics calculate how much a given point varies from the points before and after it, and can be used as a measure of how much a protein or phosphorylation site changes across a time course (31, 40, 41, 42). This analysis identifies sites with changes in stoichiometry, such as H2AX pS122 and H2AX pS140 which increase in abundance without significant changes in protein levels, as well as sites such as H2AX pS137 and PDHA1 pS232 that decrease in abundance. In contrast, sites such as LAT pS87 only change as a result of increased protein abundance (protein-normalized phosphorylation site abundance constant across the time course) (Supplemental Fig. S5*C*). This analysis also identifies phosphorylation sites whose abundances are dynamic across the time course, but do not have significant fold change between 0 and 24 h (Supplemental Fig. S5*A*). An example of phosphorylation of this type is STIM1 pS575. Phosphorylation at this site inhibits the interaction of STIM1 with EB1, resulting in reduced activity and increased cell motility (43, 44, 45). We observed STIM1 pS575 to be elevated at 4, 6, and 8 h after activation, but not at 24 h; additional work is required to investigate how this change in phosphorylation may alter cell motility during activation. We also performed this analysis on the changes in thermal shift across the time course (Supplemental Fig. S5*D*). Phosphorylation sites that were regulated in both abundance and thermal shift across the time course were enriched for the Cell Cycle KEGG pathway, as well as the GO terms double-strand break repair and DNA recombination (Supplemental Fig. S5, *E* and *F*). To ask which kinases might be driving the phosphoproteome changes in both abundance and thermal shift, we performed a kinase motif enrichment analysis (33); perhaps unsurprisingly the CDK1-6 motifs were very prominent amongst these phosphorylation sites, but we also observed motifs of unexpected kinases such as ULK1-2, involved in mitophagy signaling, as well as IRE1-2, involved in the unfolded protein response (Fig. 3*D*).

### Functional Regulation of TCR Signaling Phosphorylation Sites

**Fig. 4:**
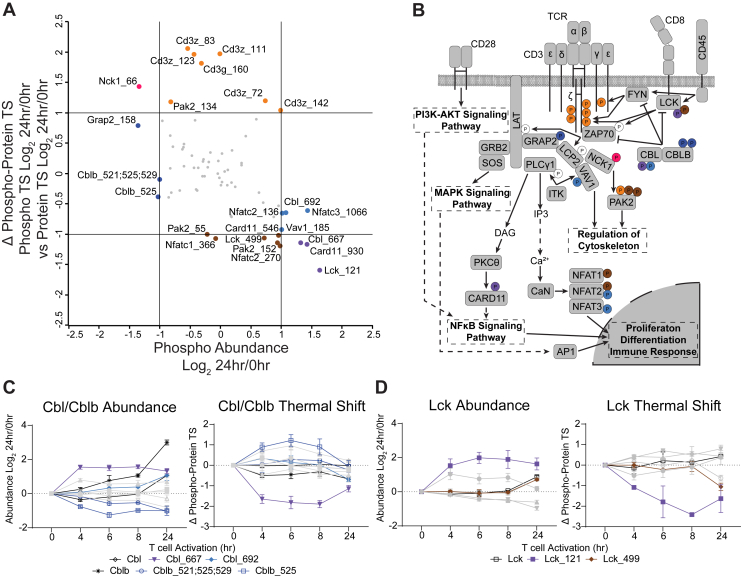
**PITSA Reveals Thermal Stability Regulation of the TCR Signaling Pathway.***A*, a comparison of the difference between the phosphorylation site and protein thermal shift (Δ Phospho-Protein TS) *versus* phosphorylation site abundance for the TCR signaling pathway (derived from Fig. 3*B*). Lines represent 2-fold change. *B*, a schematic of the TCR signaling pathway, with regulated phosphorylation sites from (*A*) indicated; colors coordinate with categories from (*A*). Phosphorylation site abundance and thermal shift for CBL and CBLB (*C*) as well as LCK (*D*). Values are the mean Log_2_ fold change of the indicated timepoint *versus* 0 h of n = 2 to 4 replicates. Error bars (*C* and *D*) represent SEM.

Within the TCR signaling pathway, we observed established phosphorylation events on known pathway members, such as CD3γ/ζ, CBL/CBLB, and LCK. Many of these sites were not altered in either the abundance or thermal shift dimensions in this experimental paradigm (Fig. 4, *A* and *B*). Of phosphorylation sites that were altered, those on TCR receptor complex members CD3ζ and CD3γ were interesting. We did not observe huge increases in phosphorylation at these sites (probably due to this signaling being resolved quickly, within 5–30 min of TCR engagement (6)). However, when phosphorylated at these sites, CD3 proteins are more thermally stable, perhaps as a result of the formation of signaling complexes from SH2-pY interactions. The differential regulation of CBL and CBLB is also intriguing; these proteins are E3 ubiquitin ligases that negatively regulate TCR signaling by degrading key activating kinases such as LCK, FYN, and ZAP70 (Fig. 4*B*) (46, 47). While the abundance of CBLB increased earlier and more significantly than CBL, both CBL and CBLB seem to be regulated by phosphorylation, but in opposite directions; CBL pS667 increases in abundance by 4 h but decreases in thermal stability, while CBLB pS525 decreases in abundance, bottoming out by 6 h, yet is correlated with an increase in thermal stability (Fig. 4*C*). A similar observation was made for LCK pS121, with increased phosphorylation by 4h of activation, but a significant decrease in the thermal stability of proteins with this phosphorylation site (Fig. 4*D*). LCK is known to be activated by dephosphorylation of pY sites by CD45 (48); why a pS site is increased several hours after TCR activation is unknown, but may be part of a negative feedback mechanism. Indeed, LCK pS121 fits the consensus motif of PRKD1, which is downstream of PKC activation and known to be involved in T cell activation (49, 50). Further investigation is required to characterize the functional roles of these phosphorylation sites.

### Regulation of DNA Damage Repair Pathways by Phosphorylation

**Fig. 5:**
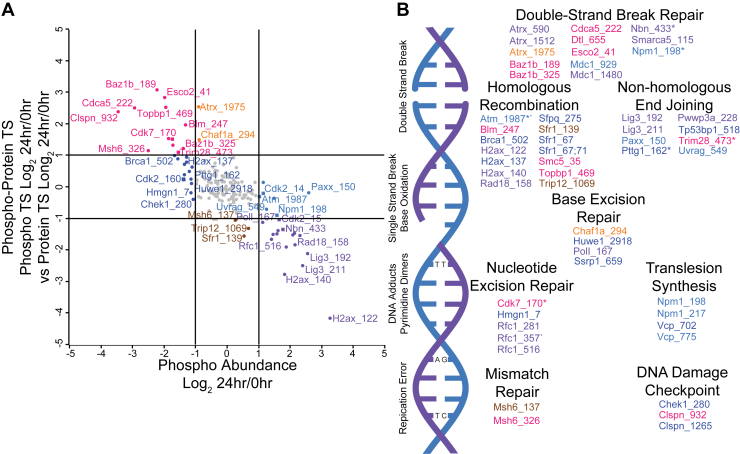
**PITSA Identifies Regulatory Phosphorylation Sites in DNA Damage Repair Pathways During T Cell Activation.***A*, a comparison of the difference between the phosphorylation site and protein thermal shift (Δ Phospho-Protein TS) *versus* phosphorylation site abundance for DNA Damage Repair pathways (derived from Fig. 3*B*). Lines represent 2-fold change, square points indicate sites with annotated functions in PhosphoSitePlus. *B*, phosphorylation sites regulating proteins in various DNA Damage Repair pathways. Colors coordinate with (*A*), *asterisks* indicate phosphorylation sites with annotated function in PhosphoSitePlus.

In addition to TCR signaling, we used PITSA to investigate additional pathways playing a role in T cell activation. Several studies have observed that T cells are particularly sensitive to DNA damage and that manipulation of the DNA damage response may be a potential therapeutic avenue for immune-related diseases (51, 52, 53). As previously noted, ITSA indicated that the PolD complex in activating T cells was in its trimeric, DNA editing form, however, we also observed a striking number of phosphorylation sites on proteins involved in DNA damage repair (DDR) pathways, many of which are altered in both phosphorylation site abundance and thermal stability (Fig. 5, *A* and *B*). While a few of these sites have functional annotation, such as NBN pS433 which regulates DNA repair pathway choice (in this case phosphorylation of S433 favors the classical non-homologous end joining pathway over alternative non-homologous end joining by inhibiting interaction with TRF2) (54), the vast majority of these sites have not been studied. Current investigation is ongoing to determine how these phosphorylation sites regulate protein function, and how this regulation may be involved in the sensitivity of T cells to DNA damage.

### CDK Substrates Have Decreased Thermal Stability

**Fig. 6:**
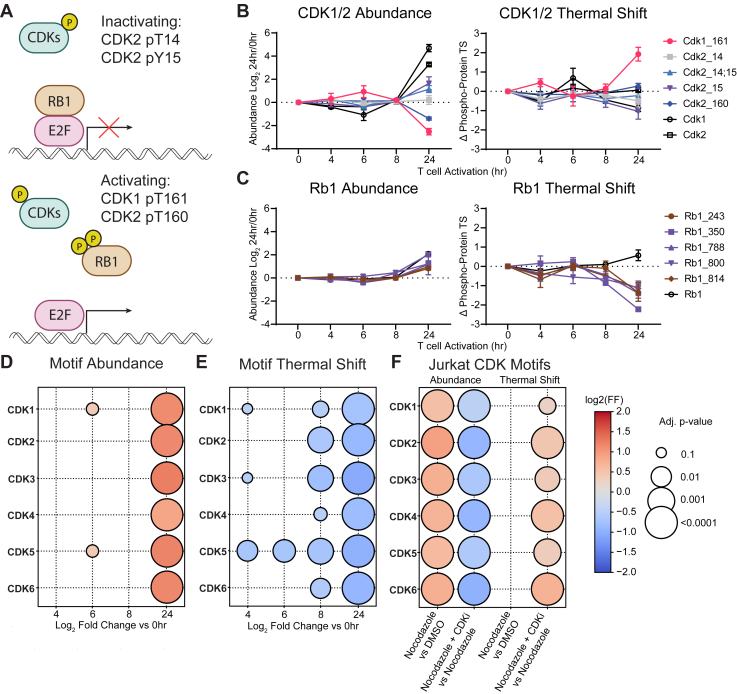
**Functional Consequences of CDK Kinase Activity Identified by PITSA.***A*, schematic of RB1 regulation by CDKs. Phosphorylation site abundance and thermal stability for CDK1 and 2 (*B*) as well as RB1 (*C*). Values are the mean Log_2_ fold change of the indicated timepoint *versus* 0 h of n = 2 to 4 replicates. Error bars (*C* and *D*) represent SEM. Motif analysis showing the abundance (*D*) and thermal shift (*E*) of predicted CDK1-6 substrates. Color indicates Log_2_ fold change *versus* 0 h, size indicates adjusted *p*-value. *F*, motif analysis showing the abundance and thermal shift of predicted CDK1-6 substrates in Jurkat cells treated with DMSO, Nocodazole, or Nocodazole + CDK inhibitors. The color indicates a Log_2_ fold change of the indicated comparison (Nocodazole *versus* DMSO or Nocodazole + CDKi vs Nocodazole), and size indicates an adjusted *p*-value.

In concordance with pathway enrichments indicating cell cycle activity, we also observed phosphorylation on canonical cell cycle proteins, including CDK1 and 2, as well as their substrate RB1. When CDKs are inactive, RB1 binds E2F and prevents transcription of cell cycle genes; however, when CDKs are activated, they phosphorylate RB1 on multiple residues, disrupting the RB1-E2F interaction and leading to transcription (Fig. 6*A*) (9, 10). CDK1 and 2 are activated by phosphorylation sites on T161 and T160, respectively, while CDK2 is inactivated by phosphorylation at T14 and Y15 (55, 56, 57, 58). Our data indicate that by 24 h, CDK1 and 2 were beginning to turn off, as activating sites were decreasing in abundance and inactivating sites were increasing. Interestingly, the CDK1 pT161 site, although decreasing in abundance, showed an increase in thermal stability, while inactivating sites decreased the kinases’ thermal stability (Fig. 6*B*). Supporting this finding, RB1 showed an increase in phosphorylation abundance at 24 h, and when phosphorylated the protein had reduced thermal stability, presumably from loss of the protein-protein interaction with E2F (Fig. 6*C*). A kinase motif enrichment analysis (33) revealed that at 24 h of activation, there was an increase in predicted CDK substrate abundance but a decrease in predicted CDK substrate motif thermal stability (Fig. 6, *D* and *E* and Supplemental Fig. S6, *A* and *B*). This result suggests that CDK phosphorylation of its substrates resulting in protein complex disruption is applicable to a broad class of its substrates, in a similar manner to RB1.

To probe this idea, we treated Jurkat T cells with nocodozole to stall the cell cycle in a regime of high CDK activity (59), and compared CDK kinase activity to DMSO control, or to nocodazole plus CDK inhibitor treatment. As expected, nocodazole treatment increased CDK substrate motif phosphorylation, which was reduced by the addition of CDK inhibitor (Fig. 6*F*, Supplemental Fig. S7, and Supplemental Dataset S2). Although we did not observe a decrease in predicted CDK substrate thermal stability upon nocodazole treatment, phosphorylation site thermal stability was increased upon the addition of CDK inhibitor to nocodazole treatment (Fig. 6*F* and Supplemental Fig. S7). In sum, these data support an association between CDK phosphorylation and decreased thermal stability of known and predicted CDK substrates.

## Discussion

Since the advent of thermal stability methods, the potential to functionalize protein PTMs has become apparent. However, using thermal proteome profiling/cellular thermal shift assay (TPP/CETSA) methods, where each TMT channel measured an individual temperature, has significant throughput limitations (60, 61, 62), while proteome integral solubility alteration (PISA) methods, where equal volumes for each temperature are combined to experimentally integrate under the melting curve, are limited to experimental regimes where protein abundance is static (8, 13). By combining the abundance and thermal stability measurements into a single TMT-plex, we are able to normalize the thermal stability values to the protein abundance, thus maintaining the throughput benefits of PISA while having the experimental flexibility of TPP/CETSA. PITSA has the additional benefit of being able to gain insight from both abundance and thermal stability measurements, providing deeper insight than previous studies investigating phosphorylation using thermal stability methods (12, 15, 16, 17). This insight has proven especially useful in interpreting the observed changes, allowing for the categorization of protein and phosphorylation site behavior into biologically meaningful groups (such as proteins that are only altered in the thermal shift dimension, which are enriched in chromatin-related proteins).

For example, a previous study investigating changes in thermal stability during the cell cycle (10) observed an increase in retinoblastoma (RB1) thermal stability after G1 exit, which is somewhat corroborated by our data, although the magnitude of increased stability is not as large in this study (Fig. 5*C*). The authors postulated that this stabilization was due to reorganization of disordered regions of the protein when phosphorylated. However, our data show that when phosphorylated, RB1 is thermally destabilized, which is correlated with its dissociation from E2F. While the question of how this observation of decreased phosphorylation site thermal stability can be reconciled with the slight increase in thermal stability of the bulk RB1 protein will require additional investigation, in general, our data support the previous conclusion that the cell cycle drives significant changes in protein function (9, 10, 12), and by using PITSA to investigate protein and phosphorylation site abundance as well as thermal stability, we are able to identify areas of protein regulation that are incompletely understood and begin to generate new models and hypotheses to test those models.

Recently, a forward genetic screen that utilized CRISPR base editors to screen for amino acid substitutions that altered human T cell activation (63) helps corroborate the functional relevance of many of the phosphorylation sites with altered thermal stability in this study. For example, LCK S121 mutation resulted in a decrease in PD1 expression, while LCK T499 mutation (although potentially any residue from 496-499) resulted in increased expression of CD25, TNFα, and INFγ. Unfortunately, because of the limitations of CRISPR targeting, not every phosphorylation site identified in this study was screened (such as those on Cbl S667 and Cbl S692), however, future studies may utilize PITSA and CRISPR base editor screening as complimentary methods to identify functionally relevant phosphorylation sites.

While PITSA proved useful in delineating functional phosphorylation sites, it appears the analysis whereby we identify functional phosphorylation sites by the difference in thermal shift to the bulk protein only identifies functional phosphorylation sites with relatively low occupancy. In regimes where phosphorylation site occupancy is high, the phosphorylated form of the protein may dictate the bulk protein thermal stability, resulting in no difference between the phosphorylation site and bulk protein thermal stability. This observation may explain why we did not observe significant thermal shifts for predicted 14-3-3 binding sites.

In summary, we have demonstrated that PITSA is a proteome thermal stability method that is amenable to a variety of experimental designs by balancing throughput and the ability to investigate regimes with dynamic protein abundance. While in this study PITSA was applied to T cell activation, generating an unprecedented view of the relationship between protein thermal stability, phosphorylation, and protein-protein interaction networks, this method will be employed to investigate many other biological processes to improve understanding of protein function and regulation.

## Data Availability

The mass spectrometry proteomics data have been deposited to the ProteomeXchange Consortium *via* the PRIDE partner repository with the dataset identifiers PXD050772 (Identifying Functionally Relevant Proteins and Phosphorylation Sites During T Cell Activation Using an Integrated Thermal Shift Assay) and PXD050445 (Investigation of CDK Substrate Thermal Stability in Jurkat T Cells).

## Supporting information

This article contains supporting information.

## Conflict of interests

The authors declare the following financial interests/personal relationships which may be considered as potential competing interests:

S. P. G. is a member of the scientific advisory boards of Cell Signaling Technologies and ThermoFisher Scientific. L. C. C. is a founder and member of the board of directors of Agios Pharmaceuticals and is a founder and receives research support from Petra Pharmaceuticals; is listed as an inventor on a patent (WO2019232403A1, Weill Cornell Medicine) for combination therapy for PI3K-associated disease or disorder, and the identification of therapeutic interventions to improve response to PI3K inhibitors for cancer treatment; is a co-founder and shareholder in Faeth Therapeutics; has equity in and consults for Cell Signaling Technologies, Volastra, Larkspur and 1 Base Pharmaceuticals; and consults for Loxo-Lilly. J. L. J has received consulting fees from Scorpion Therapeutics and Volastra Therapeutics. T. M. Y.-B. is a co-founder of DeStroke.
