# Supplementary material for: Profiling Proteins and Phosphorylation Sites During T Cell Activation Using an Integrated Thermal Shift Assay
Source: Mol Cell Proteomics. 2024 Jun 15;23(7):100801. doi: 10.1016/j.mcpro.2024.100801 (PMC11298636; doi:10.1016/j.mcpro.2024.100801)
Supplement: Supplemental Figures and Tables [file mmc3.pdf]

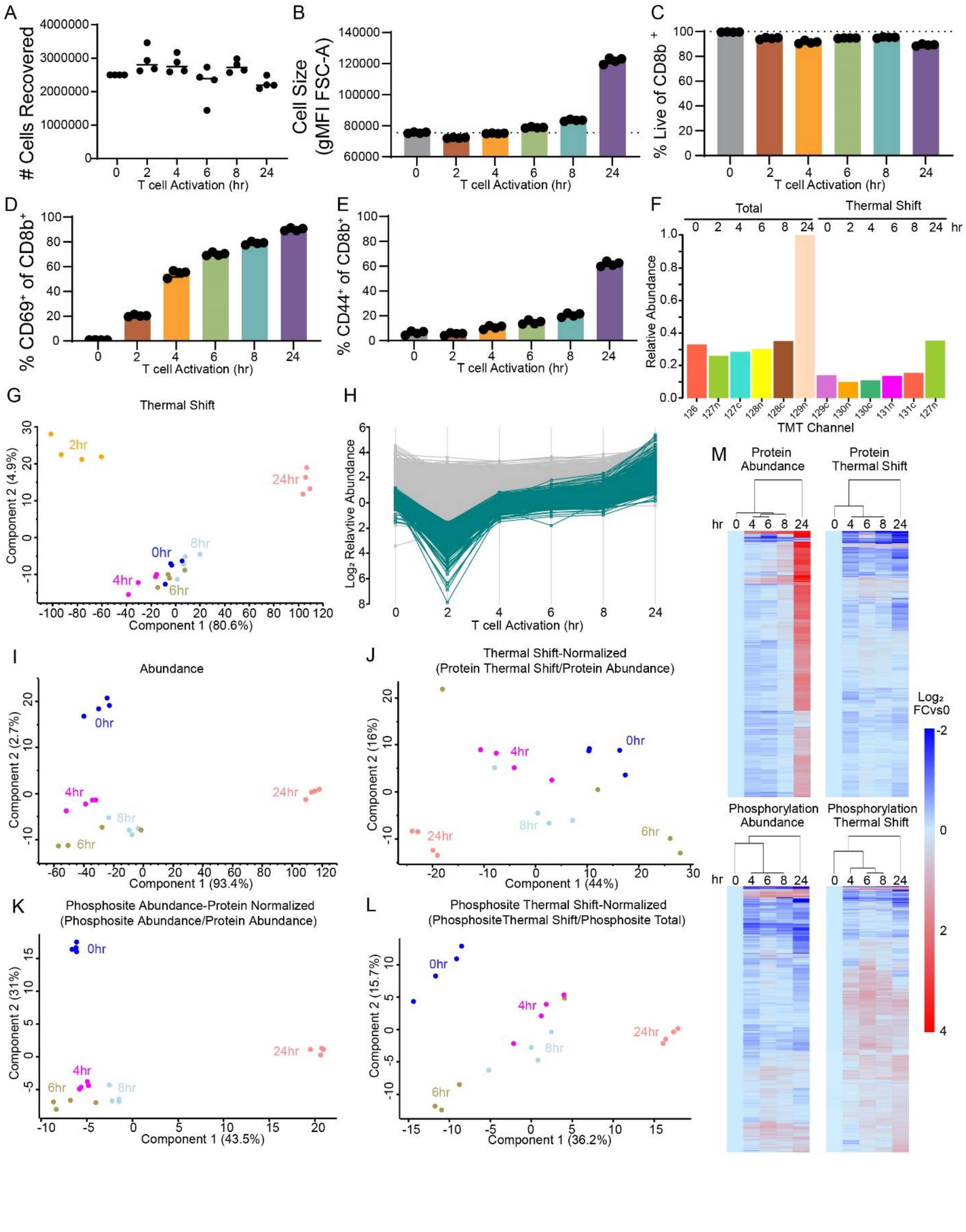
A
Cells Recovered
T cell Activation (hr)
B
Cell Size (gMFI FSC-A)
T cell Activation (hr)
C
% Live of CD8b+
T cell Activation (hr)
D
% CD69+ of CD8b+
T cell Activation (hr)
E
% CD44+ of CD8b+
T cell Activation (hr)
F
Total
Thermal Shift
hr
Relative Abundance
TMT Channel
G
Thermal Shift
Component 2 (4.9%)
Component 1 (80.6%)
2hr
24hr
0hr
8hr
4hr
6hr
H
Log2 Relavtive Abundance
T cell Activation (hr)
I
Abundance
Component 2 (2.7%)
Component 1 (93.4%)
J
Thermal Shift-Normalized
(Protein Thermal Shift/Protein Abundance)
Component 2 (16%)
Component 1 (44%)
K
Phosphosite Abundance-Protein Normalized
(Phosphosite Abundance/Protein Abundance)
Component 2 (31%)
Component 1 (43.5%)
L
Phosphosite Thermal Shift-Normalized
(PhosphositeThermal Shift/Phosphosite Total)
Component 2 (15.7%)
Component 1 (36.2%)
M
Protein Abundance
Protein Thermal Shift
Phosphorylation Abundance
Phosphorylation Thermal Shift
hr 0 4 6 8 24
Log2 FCvs0

Figure S1. Validation of T Cell Activation across Time Course. A) The number of cells recovered; B) cell size as measured by forward scatter; C) the percentage of live cells; D) the percentage of $CD8b^+$ cells expressing CD69 (an early activation marker); and E) the percentage of $CD8b^+$ cells expressing CD44 (a late activation marker) across the 24 hr time course of T cell activation. F) TMT relative abundance of individual time points/channels from a representative MS run, showing both total peptide abundance and thermal shift. G) PCA analysis of protein thermal shift across the time course of T cell activation, showing the 2 hr time point outlier. H) Profile plot showing the Log_2 relative abundance for protein thermal shift across the time points, showing the 2 hr time point dropping below the limit of quantitation. PCA analysis of I) protein abundance, J) normalized protein thermal shift (protein thermal shift/protein abundance), K) protein-normalized phosphorylation site abundance (phosphorylation site abundance/protein abundance), and L) normalized phosphorylation site thermal shift (phosphorylation site thermal shift/phosphorylation site abundance). M) K-means hierarchical clustering of Log_2 fold change vs 0 hr for protein abundance, protein thermal shift, phosphorylation site abundance, and phosphorylation site thermal shift.

Naïve

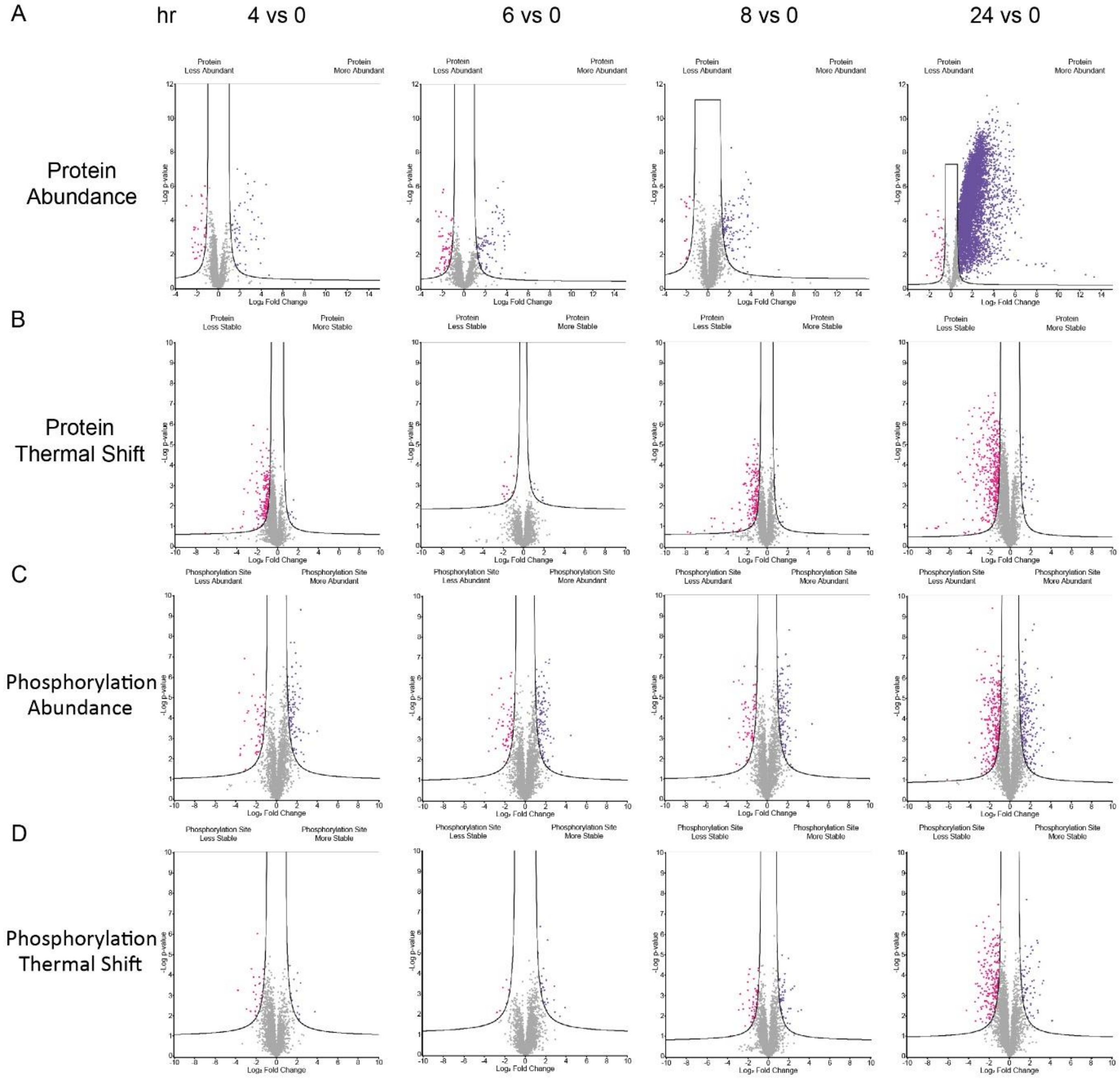

Figure S2. Time Course Comparison to 0 hr. Volcano plots comparing each time point to 0 hr for protein abundance (A), protein thermal shift (B), phosphorylation site abundance (C), and phosphorylation site thermal shift (D). X-axes represent Log_2 fold change vs 0hr; y-axes represent -Log p-value of n=4 replicates.

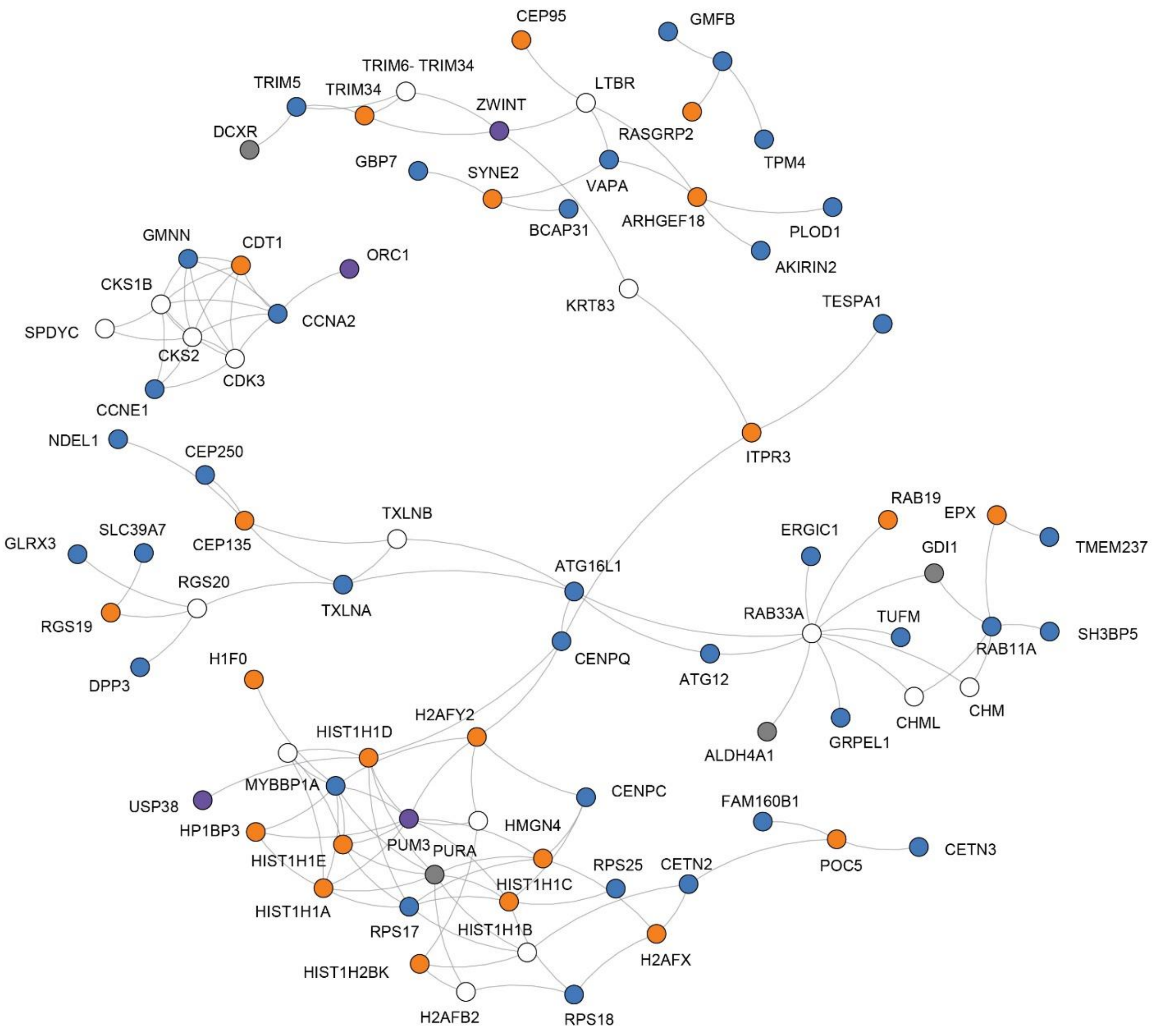

Figure S3. Network of Proteins with Increased Thermal Stability, but No Change in Abundance. Protein-protein interaction network of proteins with increased thermal stability but no change in abundance and nearest neighbors from Bioplex 3.0, coloring as in Fig. 3C.

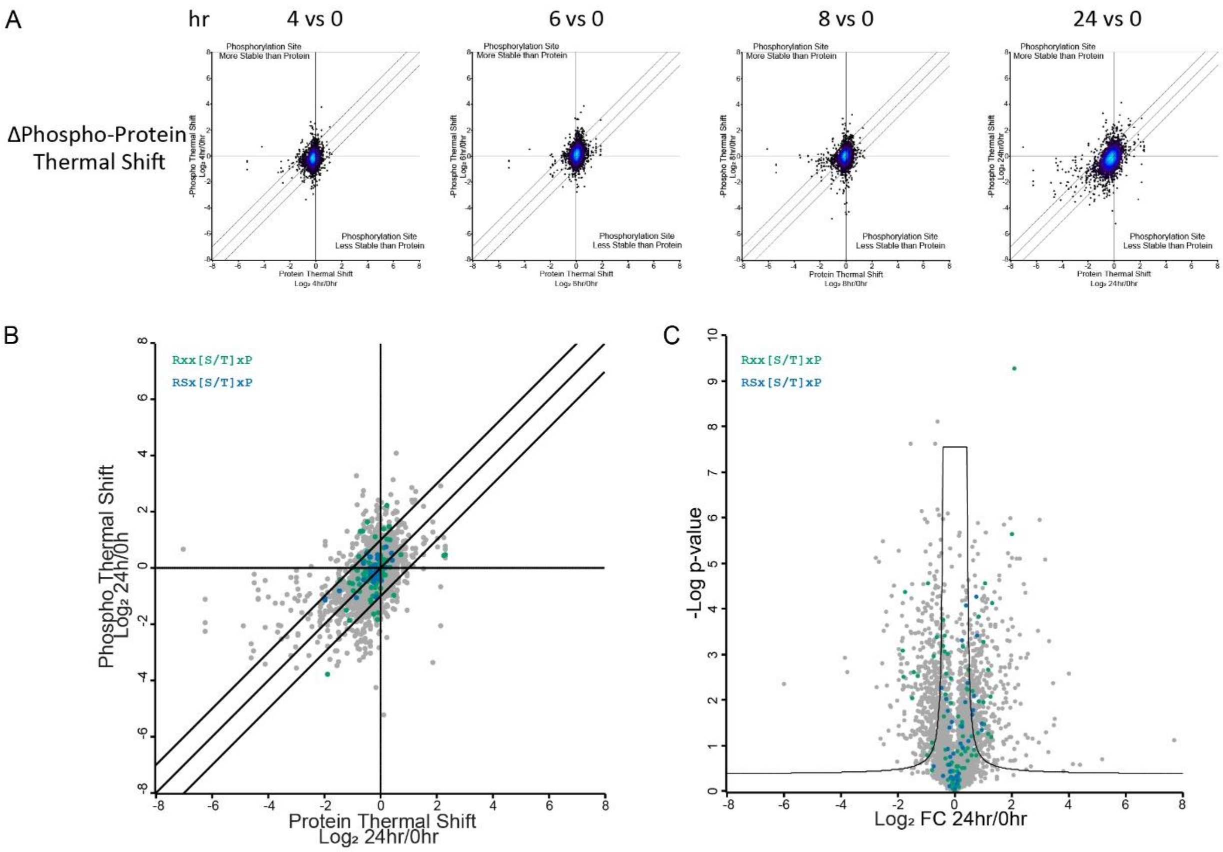

Figure S4. Identifying Putative Functionally Meaningful Phosphorylation Sites. A) A comparison of the phosphorylation site thermal shift at each time point versus 0 hr to the bulk protein thermal shift for each time point versus 0 hr. Lines indicate a 2-fold difference between protein and phosphorylation site thermal stability. Darker colors indicate reduced point density. Values are the mean Log_2 fold change of 24 hr versus 0 hr of n=4 replicates per time point. B) The phosphorylation site versus protein thermal shift at 24 hr versus 0hr (from Figure 3A), with consensus 14-3-3 binding motif phosphorylation sites highlighted. C) Volcano plot of protein-normalized phosphorylation site abundance at 24 hr versus 0 hr (from Fig S2A), with consensus 14-3-3 phosphorylation sites highlighted.

A

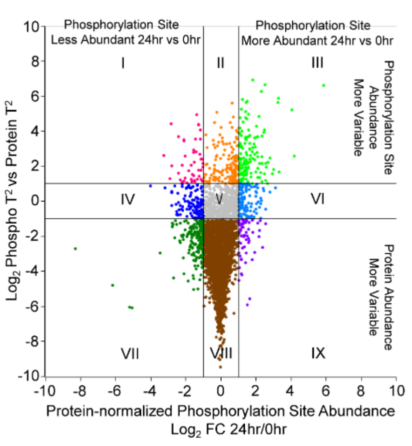

B

Category		Protein	Phospho	Stoichiometric?
I		–	↓	No
II		–	↓and↑ or ↑and↓	No
III		–	↑	No
IV		↑ or ↓	↓	No
V		–	–	Yes
VI		↑ or ↓	↑	No
VII		↑ or ↓	↓	No
VIII		↑ or ↓	↑ or ↓	Yes
IX		↑ or ↓	↑	No

C

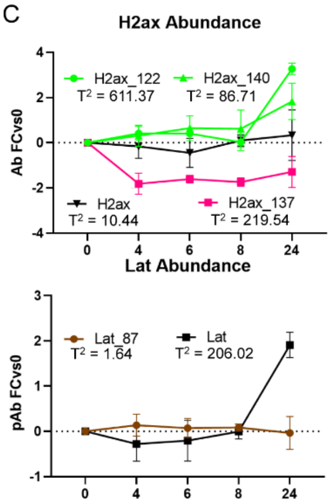

D

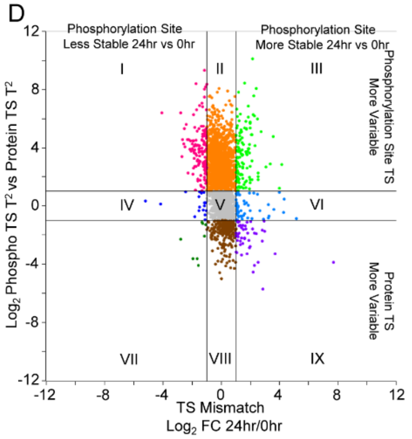

E

	Category	Enrichment	B.H. FDR
GOBP	chromatin assembly	4.846	1.68E-02
	DNA unwinding involved in replication	4.783	9.48E-03
	DNA geometric change	4.621	6.37E-04
	DNA duplex unwinding	4.621	6.37E-04
	double-strand break repair	4.266	2.20E-03
	G1/S transition of mitotic cell cycle	4.228	1.00E-02
	DNA recombination	3.534	6.37E-04
	chromatin assembly or disassembly	3.529	1.06E-02
	DNA conformation change	3.520	5.59E-04
	chromosome segregation	3.431	8.49E-03
KEGG	Cell cycle	3.353	1.11E-02

F

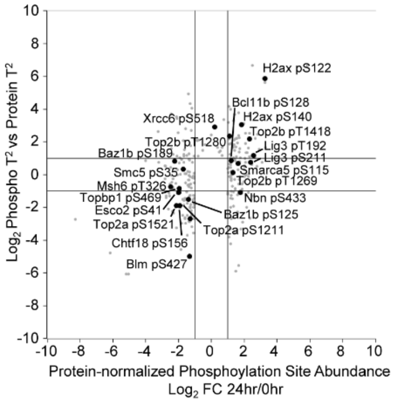

Figure S5. Identifying Stoichiometric vs Non-stoichiometric Phosphorylation Sites. A) Plot comparing the ratio of Hotelling T^2 statistics between phosphorylation abundance and protein abundance to the protein-normalized phosphorylation site abundance Log_2 fold-change at 24 hr versus 0 hr as described in Gassaway *et al.* 2021[34]. Phosphorylation site behavioral categories are denoted by roman numerals. B) Phosphorylation site behavioral category legend for (A), with the black lines representing changes in protein abundance across the time course, and colored lines representing changes in protein-normalized phosphorylation site abundance. Changes in protein or normalized phosphorylation abundance, and whether the change is stoichiometric (constant phosphorylation site abundance to protein abundance ratio) or non-stoichiometric (altered phosphorylation site abundance to protein abundance ratio). C) Representative traces of phosphorylation sites from different behavioral categories in (A). Category colors as in (A). Error bars represent S.E.M of n=4 replicates. D) Plot similar to (A), but comparing the ratio of Hotelling T^2 statistics for phosphorylation site and protein thermal shifts to the difference between phosphorylation site and protein thermal shifts. E) Category enrichment of phosphorylation sites significantly altered in both (A) and (D) (meaning sites from categories I, II, III, IV, VI, VII, and IX). F) Individual phosphorylation sites from the double-strand break repair and DNA recombination GOBP categories labelled and plotted as in (A).

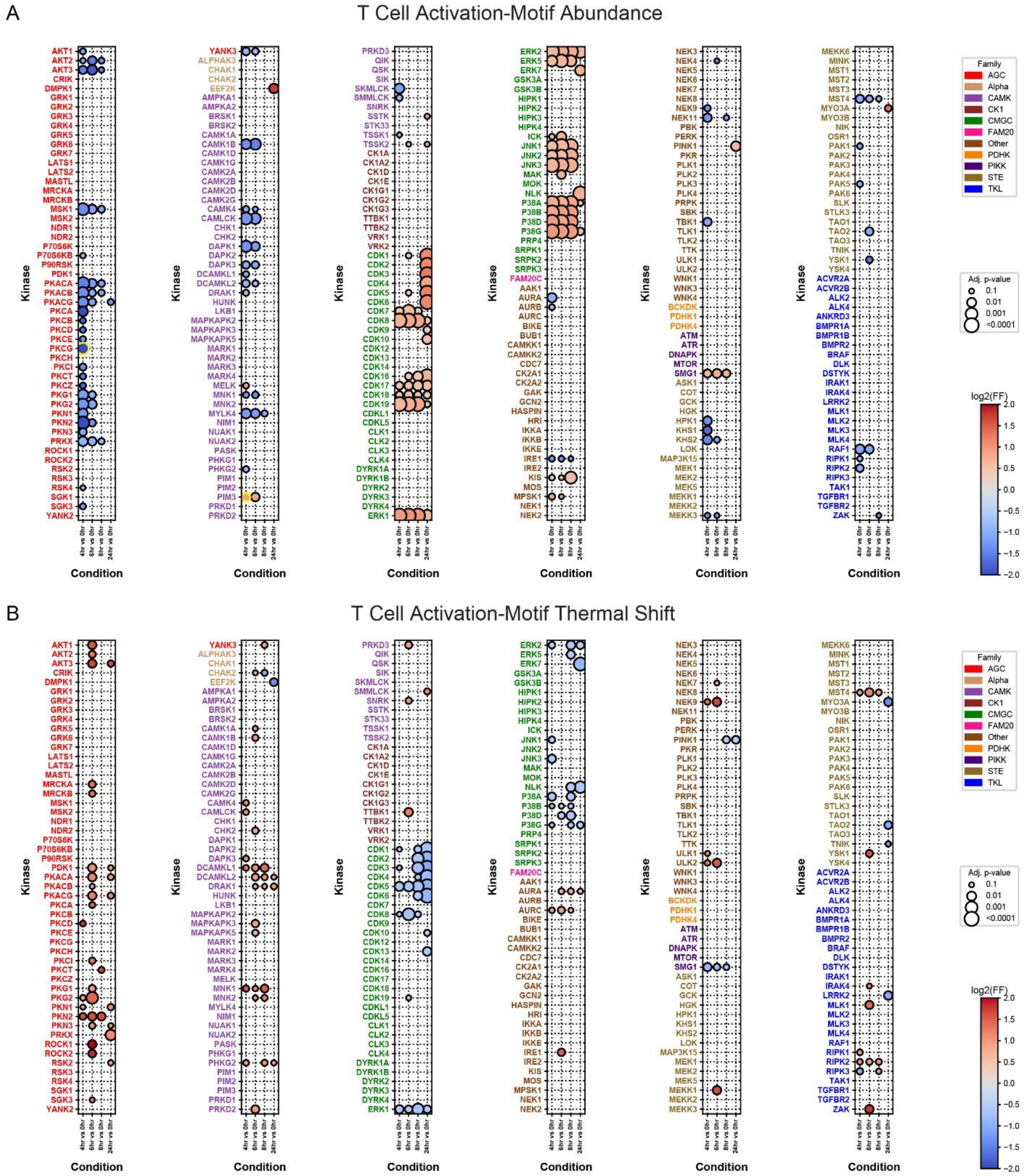

Figure S6. Kinase Motif Enrichment Analysis of T cell Activation Phosphorylation Sites. Kinase motif enrichment analysis, as published in Johnson *et al.* 2023[33], for phosphorylation site abundance (A) and thermal stability (B) values at each time point versus 0 hr. Label color indicates kinase family, circle color indicates enrichment factor, circle size indicates adjusted p-value.

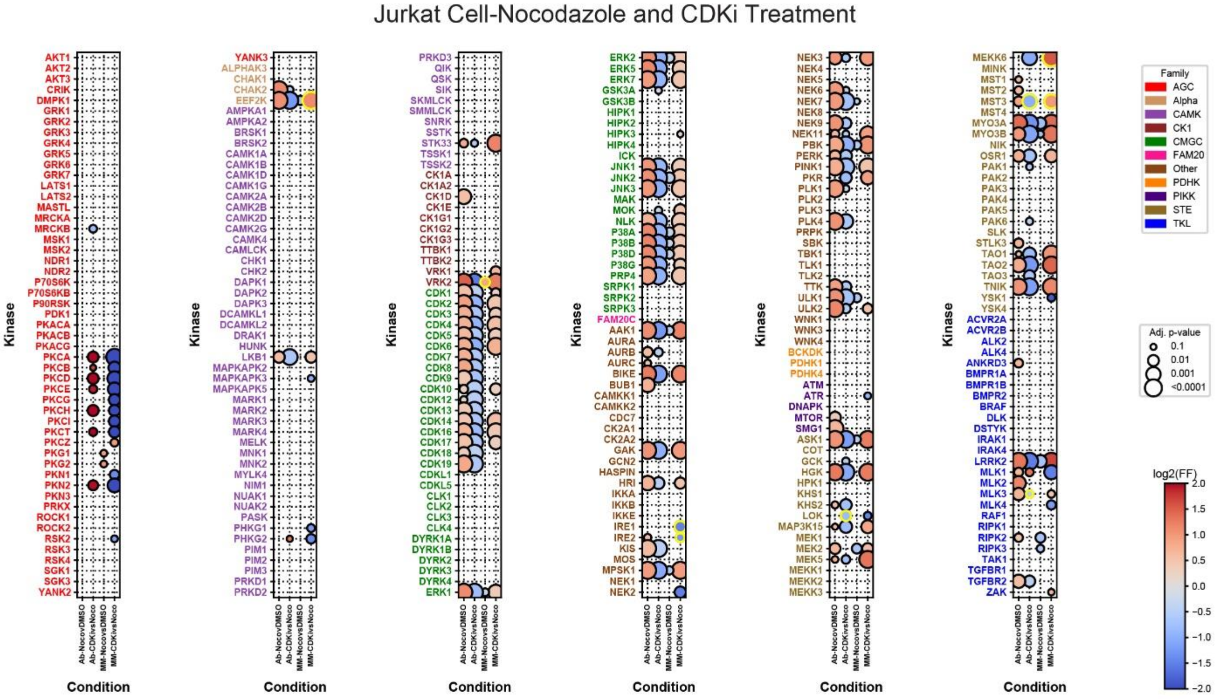

Figure S7. Kinase Motif Enrichment Analysis of Jurkat Cells. Kinase motif enrichment analysis, as published in Johnson *et al.* 2023[33], for phosphorylation site abundance and thermal stability values for Nocodazole versus DMSO treated cells and CDKi plus Nocodazole versus Nocodazole treated cells. Label color indicates kinase family, circle color indicates enrichment factor, circle size indicates adjusted p-value.

Abundance	Stability		Category	Enrichment	B.H. FDR
—	↑	GOBP	nucleosome positioning	80.26	4.56E-03
			nucleosome assembly	45.78	1.48E-12
			protein-DNA complex assembly	39.62	4.29E-12
			protein-DNA complex subunit organization	33.77	1.91E-11
			nucleosome organization	32.70	2.10E-11
			chromatin assembly or disassembly	23.41	1.54E-02
			cellular macromolecular complex assembly	6.54	5.46E-04
			chromatin organization	6.47	4.82E-05
		KEGG	Systemic lupus erythematosus	41.62	1.07E-06
		Pfam	Linker_histone	149.82	4.62E-14
			Histone	93.64	2.40E-08
↑	↓	GOBP	spindle midzone assembly	14.06	4.92E-04
			mitotic chromosome condensation	10.22	3.71E-05
			reciprocal DNA recombination	10.04	4.32E-03
			chromosome condensation	9.09	1.21E-06
			regulation of spindle organization	8.78	9.40E-03
			DNA packaging	8.43	8.14E-07
			protein localization to kinetochore	7.81	1.63E-02
			sister chromatid segregation	7.44	1.81E-04
			DNA biosynthetic process	6.69	1.59E-04
			cellular response to lipid	6.02	1.90E-02
			…		
			DNA repair	2.42	9.44E-06
		KEGG	DNA replication	4.22	1.19E-02
			Cell cycle	2.74	1.19E-02
			Pyrimidine metabolism	2.70	1.98E-02
		Corum	DNA polymerase alpha-primase complex	14.06	5.59E-03
			DNA synthesome complex	9.84	3.77E-04
		Pfam	Kinesin	9.14	1.70E-07
			Helicase_C	3.06	6.66E-03

Table S1. ITSA Category Enrichments. A Fisher's exact test was performed for the categories of protein behavior where abundance does not change over 24 hrs but thermal stability increases (orange) and where abundance increases over 24 hrs but thermal stability decreases (purple), as defined in Fig. 2C.
